# Effects of different ground segmentation methods on the accuracy of UAV-based canopy volume measurements

**DOI:** 10.3389/fpls.2024.1393592

**Published:** 2024-06-18

**Authors:** Leng Han, Zhichong Wang, Miao He, Xiongkui He

**Affiliations:** ^1^ College of Science, China Agricultural University, Beijing, China; ^2^ Centre for Chemicals Application Technology, China Agricultural University, Beijing, China; ^3^ College of Agricultural Unmanned System, China Agricultural University, Beijing, China; ^4^ Tropics and Subtropics Group, Institute of Agricultural Engineering, University of Hohenheim, Stuttgart, Germany

**Keywords:** UAV, ground segmentation, canopy volume, OTSU, RANSAC

## Abstract

The nonuniform distribution of fruit tree canopies in space poses a challenge for precision management. In recent years, with the development of Structure from Motion (SFM) technology, unmanned aerial vehicle (UAV) remote sensing has been widely used to measure canopy features in orchards to balance efficiency and accuracy. A pipeline of canopy volume measurement based on UAV remote sensing was developed, in which RGB and digital surface model (DSM) orthophotos were constructed from captured RGB images, and then the canopy was segmented using U-Net, OTSU, and RANSAC methods, and the volume was calculated. The accuracy of the segmentation and the canopy volume measurement were compared. The results show that the U-Net trained with RGB and DSM achieves the best accuracy in the segmentation task, with mean intersection of concatenation (MIoU) of 84.75% and mean pixel accuracy (MPA) of 92.58%. However, in the canopy volume estimation task, the U-Net trained with DSM only achieved the best accuracy with Root mean square error (RMSE) of 0.410 m^3^, relative root mean square error (rRMSE) of 6.40%, and mean absolute percentage error (MAPE) of 4.74%. The deep learning-based segmentation method achieved higher accuracy in both the segmentation task and the canopy volume measurement task. For canopy volumes up to 7.50 m^3^, OTSU and RANSAC achieve an RMSE of 0.521 m^3^ and 0.580 m^3^, respectively. Therefore, in the case of manually labeled datasets, the use of U-Net to segment the canopy region can achieve higher accuracy of canopy volume measurement. If it is difficult to cover the cost of data labeling, ground segmentation using partitioned OTSU can yield more accurate canopy volumes than RANSAC.

## 1 Introduction

Precise management, such as application and pruning, of the canopy is important for fruit yield and quality. Canopy volume can provide a reference for precise pesticide application and pruning. Several pesticide application models require the use of canopy volume as an input variable (Gil et al., 2013; Nan et al., 2019; Sultan Mahmud et al., 2021). However, accurate measurement of canopy volume relative to tree height is more difficult (Tsoulias et al., 2019). To accurately obtain canopy volume, traditional methods require a number of expensive manual measurements, which increases the management cost of the production process. With the development of sensor technology, LiDAR, ultrasonic sensors, and cameras are used for nondestructive and rapid measurement of canopy volume.

Terrestrial LiDAR has a wide range of applications in orchard phenology, such as canopy volume measurement and tree height measurement (Pfeiffer et al., 2018; Brede et al., 2019). Due to the long time required for a single scan and the need to scan as many locations as possible to avoid occlusions, a complete scan of a 1-ha orchard can take 3–6 days, even for an experienced team (Wilkes et al., 2017). Mobile LiDAR scanning with a real-time kinematic (RTK) receiver was developed to improve the efficiency of canopy point cloud collection (Karp et al., 2017; Wang et al., 2017; Gené-Mola et al., 2019; Mokroš et al., 2021). It has been shown that tree segmentation and canopy parameter extraction can also be achieved by LiDAR on UAV platforms, which reduce the effect of vibration and are easy to register (Yoshii et al., 2022; Yuan et al., 2022; Caras et al., 2024).

LiDAR still has a high cost compared to cameras. UAV remote sensing imagery has been widely used in the precision management of orchards (Stateras and Kalivas, 2020; Zhang et al., 2021; Pagliai et al., 2022; Sinha et al., 2022). With structure from motion (SFM) technology, three-dimensional information such as tree heights and canopy volume in orchards can be obtained using drone imagery (Mu et al., 2018; Anifantis et al., 2019; Maimaitijiang et al., 2019; Ross et al., 2022; Vélez et al., 2022; Vinci et al., 2023). For some orchards, crown diameter also can be measured (Chang et al., 2020). Leaf area index (LAI) and leaf porosity can even be obtained using multispectral images (Raj et al., 2021; Zhang et al., 2024), while crop water stress index can also be assessed to inform precision irrigation (Chang et al., 2020). Combined with computer vision technology, it can even enable fruit recognition to provide growers with yield information in the early stages of crop growth (Ariza-Sentís et al., 2023). The digital surface modeling (DSM) created by images contains the height of the crop (Zarco-Tejada et al., 2014; Yurtseven et al., 2019; Lu et al., 2021; Tunca et al., 2024).

Furthermore, to obtain the volume of the canopy, the digital terrain model (DTM) needs to be split from the DSM (Patrignani and Ochsner, 2015; Ali-Sisto et al., 2020; Ali et al., 2021), and the canopy height model (CHM) is created by taking the difference between the DSM and the DTM (Eitel et al., 2014; Walter et al., 2018). Next, canopy volume can be obtained by summing the CHM with voxel (Stovall et al., 2017; Wallace et al., 2017). For field crops, it is easier to obtain the DSM as DTM when the crop is not planted (Maimaitijiang et al., 2019). However, for orchards, accurate ground segmentation is required for canopy volume measurement tasks. Algorithms that have been developed for ground segmentation include zone thresholding methods and plane-fitting (Sithole and Vosselman, 2004; Oniga et al., 2023; Wen et al., 2023). The results of ground segmentation can significantly affect the measurement of canopy volume. Therefore, exploring different ground segmentation methods can improve the accuracy of canopy volume measurements.

This study presents a canopy volume measurement pipeline based on UAV remote sensing images, which first constructs the RGB and DSM of the target orchard, then segments the ground and canopy regions, and finally calculates the canopy volume based on the segmented masks using DSM. The effects of different segmentation algorithms on the accuracy of canopy volume measurements are also investigated.

## 2 Materials and methods

The point cloud acquired from the moving LiDAR scan was voxelated, the voxel volumes were summed, and the calculated canopy volume was taken as the true value. The RGB and DSM images acquired by the UAV are segmented into plots, and the canopy and ground section are segmented by different segmentation methods, and the volume of the canopy is calculated without the use of high-resolution DTM data. Diagram of the experimental design is shown in **Figure 1**.

**Figure 1:**
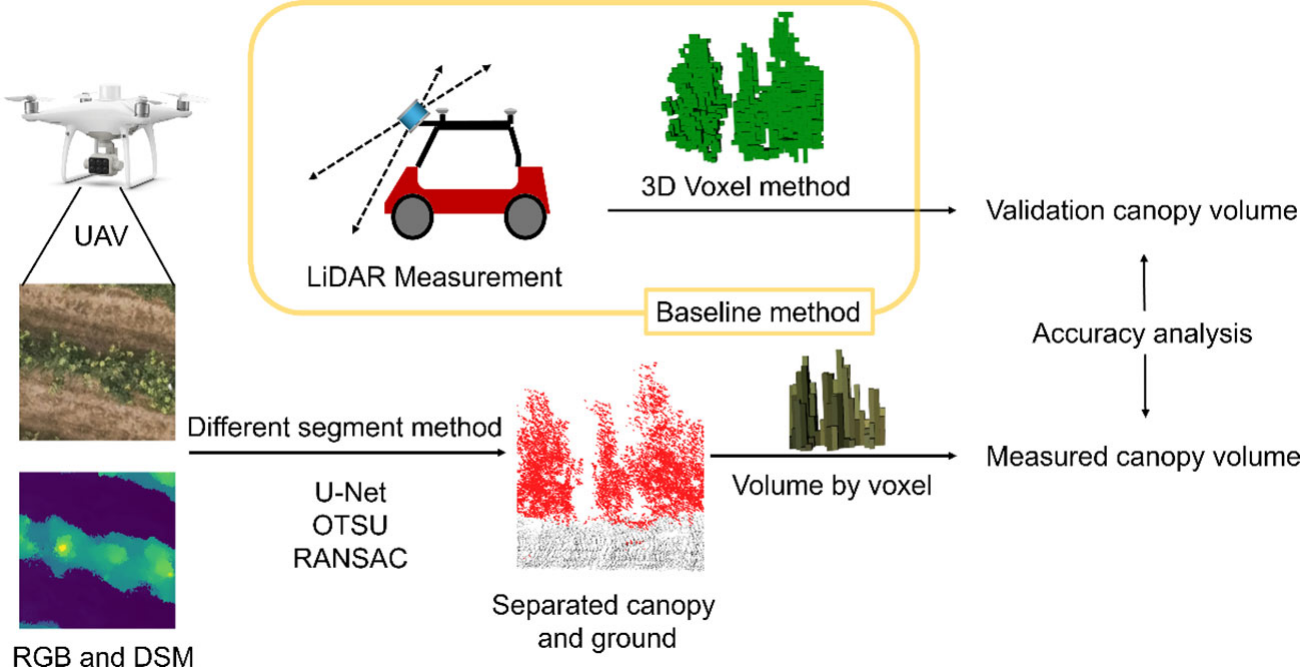
Diagram of the experimental design, LiDAR scanning as a baseline method for canopy volume measuremenlt, and comparison of the effects of different ground segmentation methods on the accuracy of canopy volume measurement.

### 2.1 Image data collection

#### 2.1.1 Data collection

**Figure 2:**
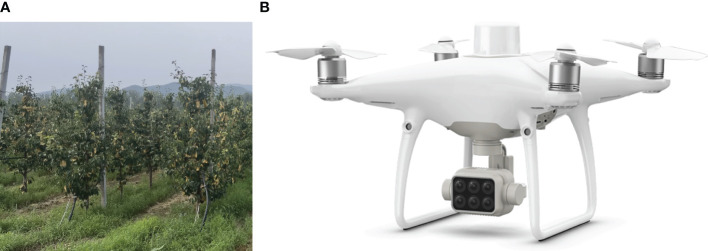
Experimental site and UAV conducting experiment. **(A)** Pear orchard with a row-to-row distance of 4.5 m. **(B)** UAV used for image acquisition.

Field experiments were conducted in a pear (*Pyrus bretschneideri* ‘Zaosuhong’) orchard in Pinggu District, Beijing (40.18°N, 116.97°E, WGS-84). The orchard covered an area of about 3 ha, and the pear trees were BBCH 91 when photographed. The trees were spaced in rows with a 4.5-m interval (**Figure 2A**) and in rows with a 1.5-m interval between trees, with an average tree height of about 4 m.

The P4 Multispectral (DJI Technology Inc., Shenzhen, China), which has one RGB camera and a multispectral camera array with five cameras covering blue, green, red, red edge, and near-infrared bands, all at 2 megapixels (MP) with global shutter, was used to acquire images (**Figure 2B**), and only its RGB channel (2 MP) was used in this experiment. The flight height was 30 m, resulting in a ground sample distance (GSD) of 0.016 m/pix. The head and side overlap were both 70%, and the images were taken at equal time intervals. Images were captured between 11:00 and 13:00 to ensure photograph quality. During the capture period, the weather was clear and windless, which eliminated the blur caused by swaying branches. During the flight, the network core service provided by Qianxun Inc. (Shanghai, China) was used to get more accurate RTK positioning.

#### 2.1.2 Image processing

The image procession utilized a workstation with Windows 10 (64-bit), 32 GB of RAM, i7–8700K, and GTX 1080Ti. The orthophotos and DSM were reconstructed with Terra (3.5.5, DJI Technology Inc., Shenzhen, China). A high reconstruction quality was selected to get high accuracy and resolution. The geographic coordinates were based on the WGS84 (EPSG: 4326) coordinate system in this study. As the P4M is equipped with an RTK receiver and the manufacturer supports phase-free control point technology, no ground image control point was set during the experiment.

### 2.2 UAV-based canopy volume measurements

The DSM of an orchard can be utilized to calculate the canopy volume (Mahmud et al., 2023). First, a segmentation operation was performed to extract the mask of the canopy region. Next, the volume of the region between the canopy and the ground was calculated as the final measured canopy volume. In this study, a U-Net-based deep learning method, grid-based OTSU, and RANSAC methods were used to segment the canopy, and the accuracy of different segment methods was compared. The orchard is situated on a gentle hillside, resulting in the ground in the orchard not being on the same plane. To avoid misclassification in the ground segmentation, it is important to segment the elevation data of the orchard area separately. In this study, the orchard was divided into multiple 4.5 m times 4.5 m plots. The ground within each plot was considered to be in one plane.

#### 2.2.1 Deep-learning-based ground segmentation methods

**Figure 3:**
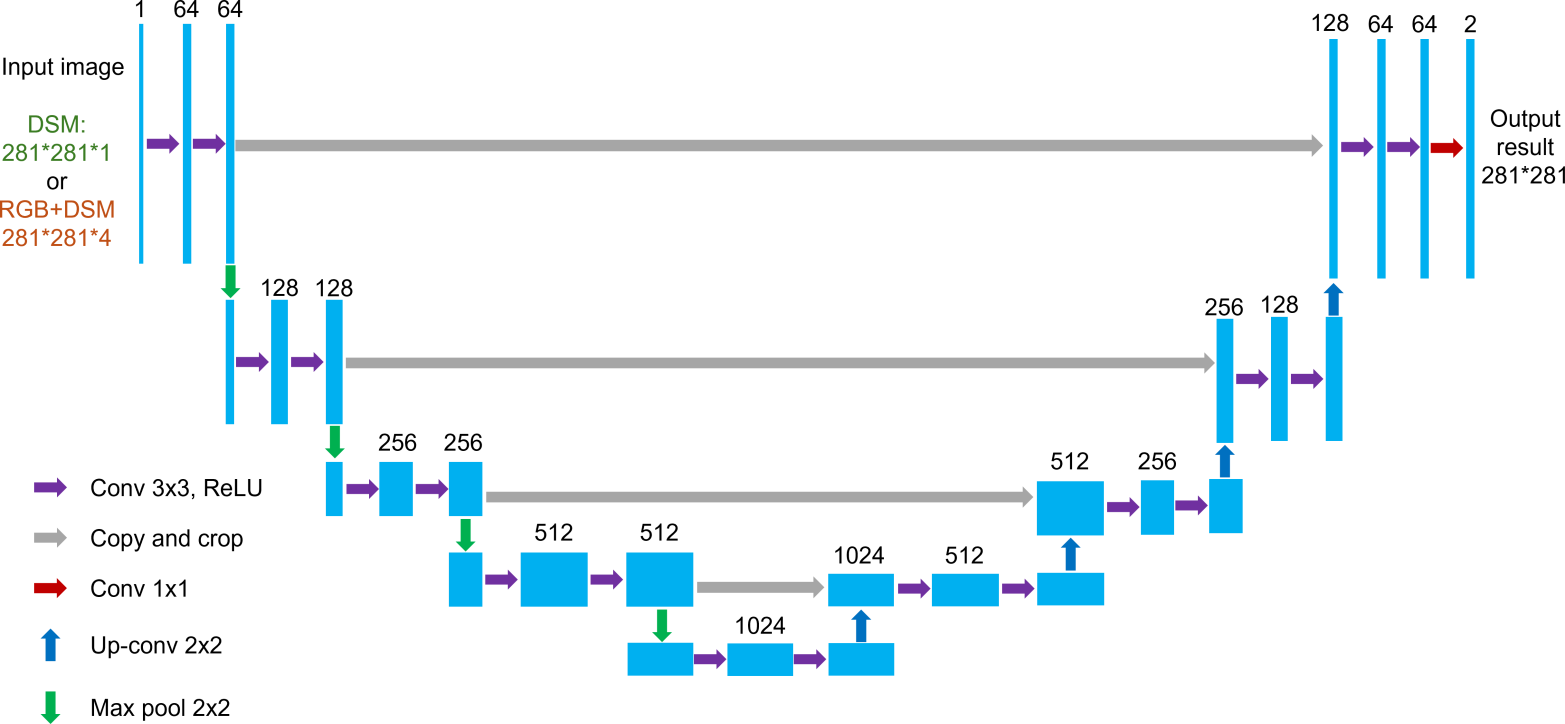
U-Net network structure used in the study. Blue boxes correspond to multichannel feature maps with a number of channels marked on the top of each box. Conv, convolution; up-conv, upconvolution; max pool, max pooling with the size of the convolution kernel.

U-Net (Ronneberger et al., 2015) is a widely used deep learning network in remote sensing for efficient semantic segmentation of input images through an encoder and decoder. The classical U-Net can obtain fast segmentation results on smaller datasets with a lightweight structure. In this study, the classical U-Net was directly used, containing 31 million parameters, and the inputs were a four-channel image of 281 pixels times 281 pixels (RGB and DSM) or a single-channel image of 281 pixels times 281 pixels (DSM) for training, and the outputs were the segmented masked images (**Figure 3**).

**Figure 4:**
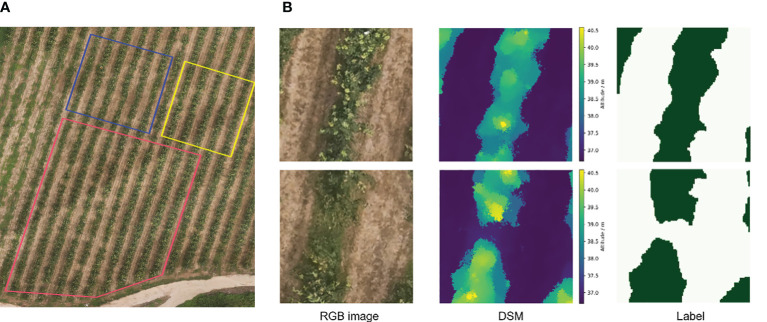
Sampling region of the data in training and the RGB images, DSM images, and corresponding labels used. **(A)** The red subfield is the random sampling region for the training set, and the blue and yellow are the test and validation sets, respectively. **(B)** Sampled training images and corresponding labels with a total of four channels of RGB and DSM were fed into the network, where the dark-green color in the labels are the canopy and the light-green-colored regions are the ground.

Three subfields from the orthophoto and the corresponding labeled files were divided as the sampling regions for the training set, validation set, and test set, with the number of samples being 400, 50, and 100, respectively (**Figure 4**). The validation set was used to adjust the epoch, batch size, and learning rate. Labels were created by an open-source annotation tool called labelme (V5.2.1).

#### 2.2.2 OTSU-based ground segmentation method

**Figure 5:**
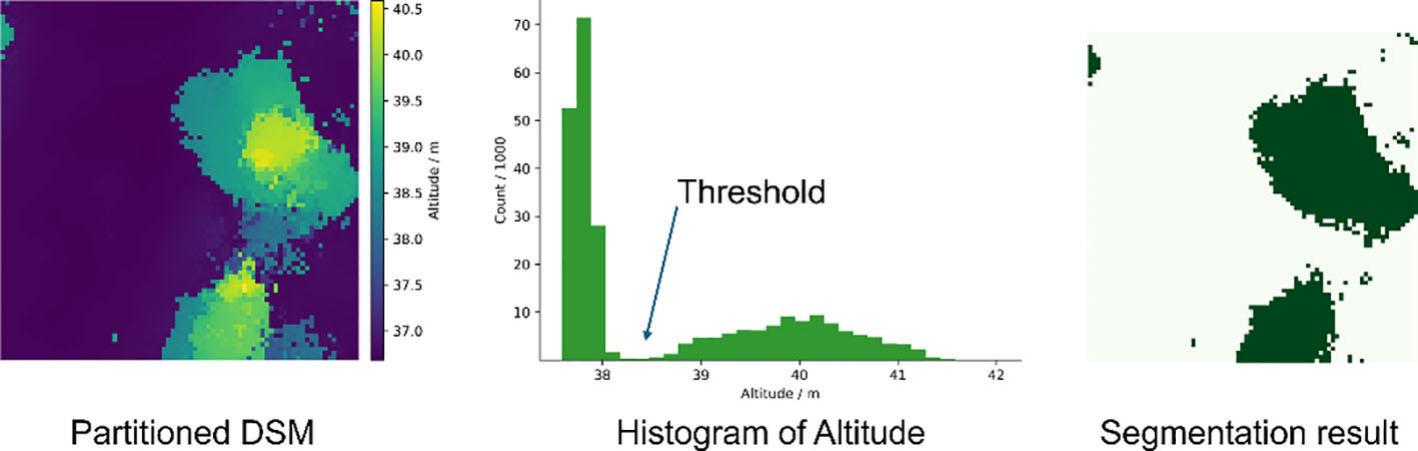
Segmentation thresholds for DSM obtained by OTSU and a mask of the canopy region, where dark green is the canopy region and light green is the ground.

The OTSU threshold segmentation method is widely used in the field of remote sensing. The values of the background and the target have different distributions, and the OTSU method selects the threshold corresponding to when the value of the interclass variance is taken to be the maximum as the optimal threshold. The points belonging to the ground had a low elevation, and the canopy points had a higher elevation in the distribution histogram of DSM, showing two peaks in the histogram. The OTSU method was used to automatically find the threshold value in the middle of the two peaks to maximize the interclass variance of the ground and canopy elevation distributions. Points with elevation above the threshold are categorized as canopy regions, and the rest are ground. **Figure 5** illustrates the segmentation process and the binarized mask map.

#### 2.2.3 RANSAC-based ground segmentation method

**Figure 6:**
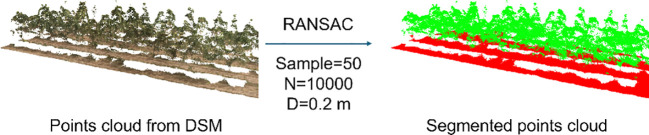
Conversion of DSM to 3D point cloud and segmentation by RANSAC.

RANSAC is an iteration-based fitting method that obtains the parameters of a model by randomly sampling the data points and calculating the probability of a successful fit. Given its good robustness, it is often used to extract planes within a point cloud. Open3D is an open-source library that supports rapid development of software that deals with 3D data. The plane fitting function therein was used in this study based on the empirical selection of 50 sampling points, 10,000 iterations (*N*), and a distance threshold of 0.2 m (*D*). The ground was fitted and split between the canopy and the ground (**Figure 6**).

#### 2.2.4 Method of tree height and canopy volume calculation

The positions of the tree base were measured using a tilt-featured RTK receiver (E500, Beijing UniStrong Science and Technology Co. Ltd., Beijing, China), and the true height of the tree was measured using a tower ruler with a height accuracy of ± 5 cm. The coordinates and tree heights of 20 trees were measured in the scanned area and used to analyze the accuracy of the tree height measurements. The tree height measured by the LiDAR or UAV was achieved by selecting points within 0.25 m from the root coordinates of the tree and calculating the difference between the maximum and minimum heights.

The volume of the canopy was accumulated from the volume of each pixel in the mask (Equation 1). The volume of the pixel was calculated by the area multiplied by the height, which was the difference between the pixel and the ground mean altitude.


(1) $$V_{canopy}=\sum^{}GSD^{2}\times \left(h_{pix}-{\bar{h}}_{ground}\right)$$

Where *V_canopy_* is the final volume, GSDis the resolution of DSM (in this study is 0.016 m/pix), *h_pix_* is the altitude of each pixel, and ${\bar{h}}_{ground}$ is the mean value of the altitude of the ground in the plot.

### 2.3 LiDAR data acquisition methods

**Figure 7:**
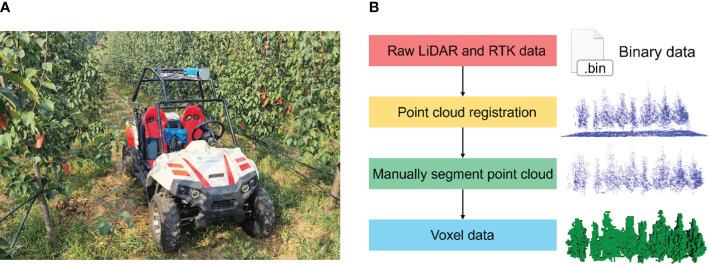
Voxel volume of the canopy obtained by LiDAR. **(A)** Vehicle collecting canopy point cloud with LiDAR and RTK rover. **(B)** Processing pipeline of point cloud data acquired by mobile laser scanning.

The true value of canopy volume is difficult to measure, and canopy calculations from moving LiDAR scans have typically been used as the true volume in previous studies (Li et al., 2017; Sultan Mahmud et al., 2021). A previously developed LiDAR-RTK fusion information acquisition system (Han et al., 2023) was used to acquire point clouds of the canopy. The LiDAR and RTK mobile stations were mounted on a frame on top of a vehicle, which allowed for smooth travel through the orchard (**Figure 7A**). Data acquisition was carried out during clear and windless hours, traveling at a speed of about 1 m/s.

The acquired point cloud was synchronized with the recorded RTK packet to obtain position and heading, and each frame of the point cloud was converted to a geographic coordinate system to obtain a complete point cloud of the scanned area. The complete point cloud was carefully removed from the ground portion manually with Meshlab software (2023.12). It was later constructed as voxel data at 0.1 m in size. Multiplying the number of voxels by the volume of a single voxel calculates the measured canopy volume (**Figure 7B**). The canopy volume calculated from the moving LiDAR-scanned point cloud was taken as the true value.

### 2.4 Statistical methods for precision evaluation

Mean intersection of concatenation (MIoU, Equation 2) and mean pixel accuracy (MPA, Equation 3) were used to evaluate the segmentation accuracy of the model. The data in the training set was segmented using OTSU and RANSAC, and the segmentation accuracy was also evaluated.


(2) $$\text{MIoU}=\left(\frac{T_{P}}{T_{P}+F_{P}+F_{N}}+\frac{T_{N}}{T_{N}+F_{P}+F_{N}}\right)/2$$

(3) $$\text{MPA}=\frac{1}{k+1}\sum_{i=0}^{k}P_{i}$$

where *T_P_* is the number of correctly classified pixels in canopy samples, *T_N_* is the number of correctly classified pixels in ground samples, *F_P_* is the number of wrongly classified pixels in canopy samples, *F_N_* is the number of incorrectly classified pixels in ground samples, and *P_i_* represents the proportion of correctly classified pixels in a different category.

In total, 50 zones of size 4.5 m * 4.5 m were selected from the scanned area of the LiDAR. The canopy volumes obtained by different methods were calculated, and the accuracy of the volumetric measurements was assessed using the moving LiDAR scans as the true values. Root mean square error (RMSE, Equation 4), relative root mean square error (rRMSE, Equation 5), and mean absolute percentage error (MAPE, Equation 6) were used to assess the error between the measured and true values.


(4) $$\text{RMSE}=\sqrt{\frac{\sum^{}{\left(V_{i}-V_{i}^{'}\right)}^{2}}{n}}$$

(5) $$\text{rRMSE}=\frac{RMSE}{\bar{V}}*100\%$$

(6) $$\text{MAPE}=\frac{100\%}{n}\sum^{}\left|\frac{V_{i}^{'}-V_{i}}{V_{i}}\right|$$

where *V_i_* is the measured volume, $V_{i}^{'}$ is the true volume (measured by moving LiDAR), *n* is the sample number, and $\bar{V}$ is the mean value of the true volume.

## 3 Result

### 3.1 Segmentation of canopy and ground

Different hyperparameter settings will have an impact on the training results. In this study, by modifying the default parameters and pretraining, the finalized hyperparameters were an epoch of 20, a batch size of 5, and a learning rate of 10^−5^. The losses of the U-Net networks trained with different input data during training are shown in **Appendix A**. Both drop faster in the first three epochs, and the loss stabilizes after 10 epochs of training.

**Figure 8:**
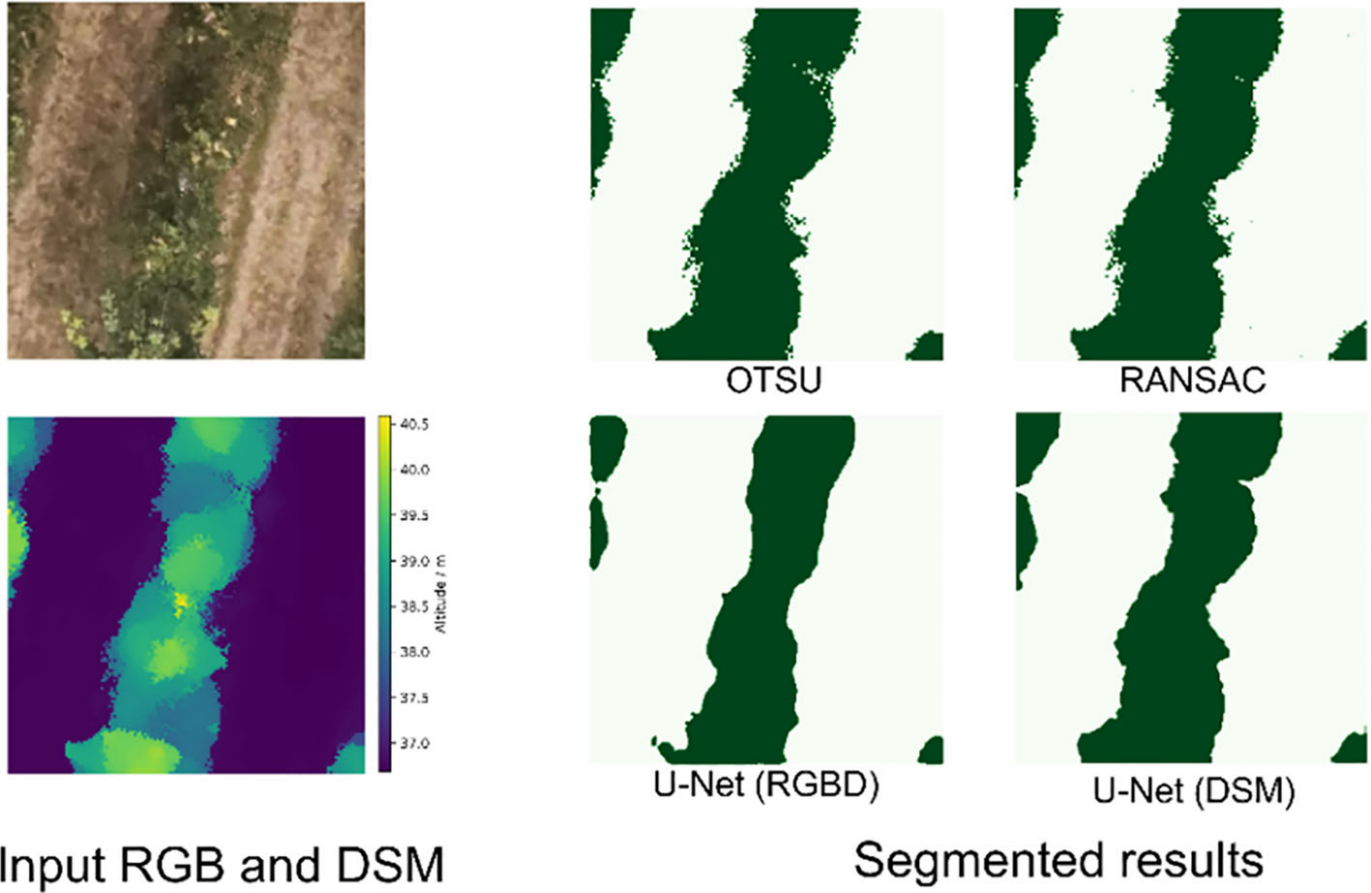
Results of different segmentation methods for tree rows: dark green for the canopy and light green for the ground.

The data in the training set were segmented by trained U-Net, OTSU, and RANSAC methods, and the example results are shown in **Figure 8**. Deep learning-based segmentation methods possess smoother edges. The neural network trained based on RGB and DSM inputs more information, and its edges will be smoother and more closely fit the actual canopy region. While OTSU and RANSAC segment by simple thresholding, the edges will have a lot of noise and reduce the recognition accuracy. In addition, RANSAC has a fixed distance threshold compared to OTSU, which will incorrectly classify the ground as a tree canopy in some areas.

**Table 1** shows the accuracy of different segmentation methods. U-Net trained by RGB images and DSM achieved the highest MIoU and MPA of 84.75% and 92.58%, respectively. RANSAC had the worst segmentation accuracy, with MIoU and MPA of 64.48% and 90.20%, respectively. The MPAs of the four methods were close to each other, indicating that the segmentation accuracies of the different methods were similar for canopy and ground level. While the difference in MIoU suggested that the different segmentation methods had different overlaps of the canopy region, the deep learning method had tidier edges, and the pixel classification accuracy would be higher at the edges. Therefore, the overlap with the correct classification was higher to get a higher MIoU. Although both the DSM-based training U-Net OTSU and RANSAC methods used only altitude data, the results of the segmentation method with deep learning were smooth, with a higher overlap with the actual canopy region.

**Table 1:** MIoU and MPA for different segmentation methods.

Method	MIoU	MPA
U-Net (RGBD)	84.75%	92.58%
U-Net (DSM)	83.37%	91.55%
OTSU	65.33%	90.56%
RANSAC	64.48%	90.20%

### 3.2 Measurement of tree height and canopy volume

**Figure 9:**
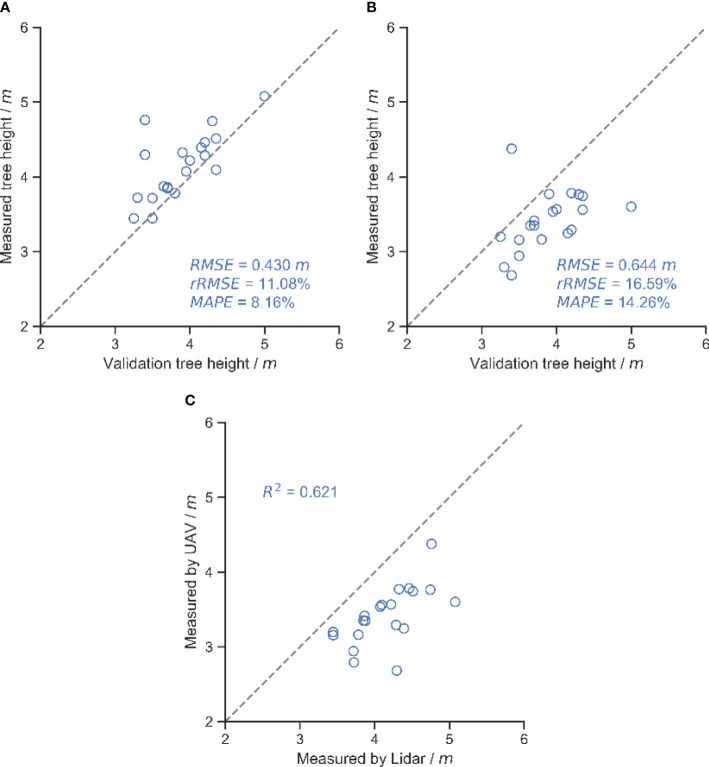
Results of different methods of measuring tree height. **(A)** Tree height measured by LiDAR. **(B)** Tree height measured by UAV. **(C)** Comparison of the results of the two methods of measuring tree height.

**Figure 9** shows the results of tree height measurements using moving LiDAR scanning and a UAV. The RMSE of the tree height measured by LiDAR was 0.430 m and MAPE was 8.16%, while the RMSE and MAPE of UAV were 0.644 m and 14.26%, respectively. The UAV showed a greater error, which was probably due to the DSM construction process with some errors. Comparing the measurements of the two methods for the same tree, it could be found that the UAV’s measurements were low.

**Figure 10:**
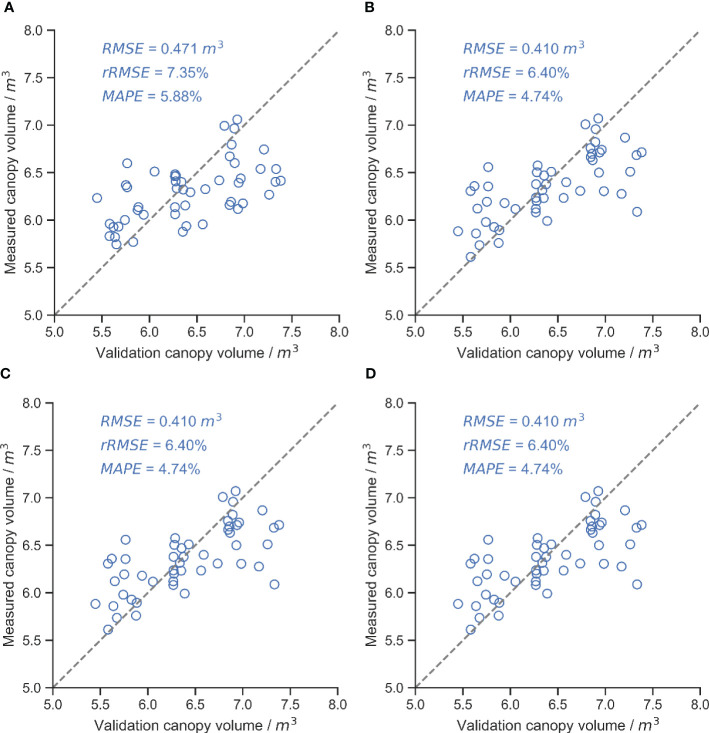
Results of canopy volume measurements for different segmentation methods, with the gray dashed line in the figure being the 1:1 line. **(A)** U-Net (RGB and DSM). **(B)** U-Net (DSM). **(C)** OTSU. **(D)** RANSAC.

**Figure 10** shows the accuracy of canopy volume measurements for different segmentation methods. U-Net trained with DSM obtained the highest canopy volume measurement accuracy with an RMSE of 0.410 m^3^. However, RANSAC segmentation had the worst canopy volume measurement accuracy, with an RMSE of 0.580 m^3^. The accuracy of deep learning-based segmentation approaches was higher than that of traditional methods, consistent with the results of segmentation accuracy. In the OTSU and RANSAC methods, the measurement accuracy of OTSU was higher than that of RANSAC.

## 4 Discussion

This study presented a pipeline for measuring canopy volume using UAVs and evaluated the impact of different ground segmentation methods on the accuracy of the measurements. The results indicated that the deep learning-based segmentation method had higher accuracy than the OTSU and RANSAC methods, whether trained with RGB and DSM or only with DSM. The U-Net model trained with an additional RGB channel input provided more color and texture information, improved the segmentation accuracy (Geirhos et al., 2018), and resulted in the highest segmentation accuracy with MIoU of 84.75%. Since the edges of manual labels were smooth, the neural network learned the feature of smooth edges, and the segmentation results do not need filtering operations.

**Figure 11:**
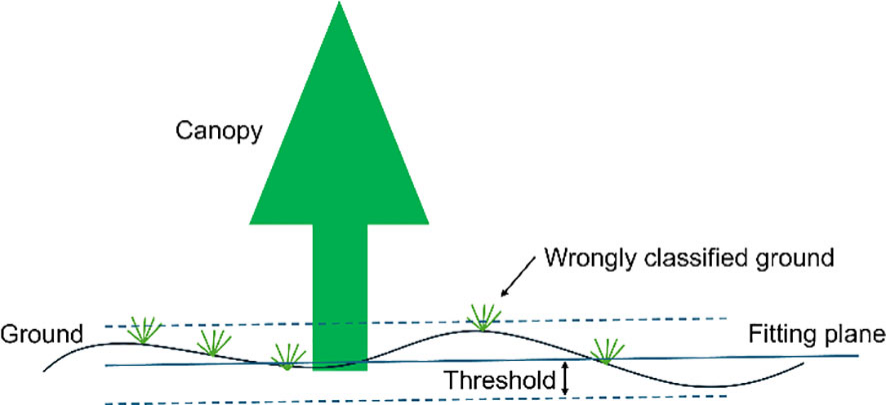
The RANSAC method incorrectly segments undulating ground and wild weeds.

Since the filtering operation on the mask image involved different filtering algorithms and hyperparameters, the output results were not filtered in this study but were directly used to calculate the canopy volume in the next step in order to evaluate the differences between the different algorithms themselves. The difference in the performance of OTSU and RANSAC at the edges might result in the identity of the masked area being larger than that manually labeled, which leads to a lower MIoU. At the same time, RANSAC might incorrectly classify a small number of ground points as a canopy, and some “pretzel-like” noise points can be seen in the ground part of the segmentation results. RANSAC incorrectly classified a small number of ground points as a canopy, and some “pretzel-like” noise points were visible in the ground portion of the segmentation result. In the DSM constructed using UAV, there were weeds in the ground part, and the ground part has a certain “thickness” due to the accuracy of the SFM method, and RANSAC uses a fixed threshold (0.2 m in this study) to misclassify some of the ground points as canopy (**Figure 11**). This might be the reason why RANSAC has the worst accuracy in segmentation and canopy volume measurements.

LiDAR tended to give higher results compared to manually measured true tree heights due to the fact that the LiDAR-equipped vehicle had a slight wobble when traveling, leading to incorrect measurements of tree heights (Han et al., 2023). In contrast, the tree heights measured in the UAV-based constructed DMS will be low. This might be due to the small area of the treetops, which makes it difficult to recognize the features when constructing the DSM, thus resulting in the highest point of the tree canopy not being correctly identified. Considering the difference in measurement efficiency, it is possible to measure tree height using a UAV.

**Figure 12:**
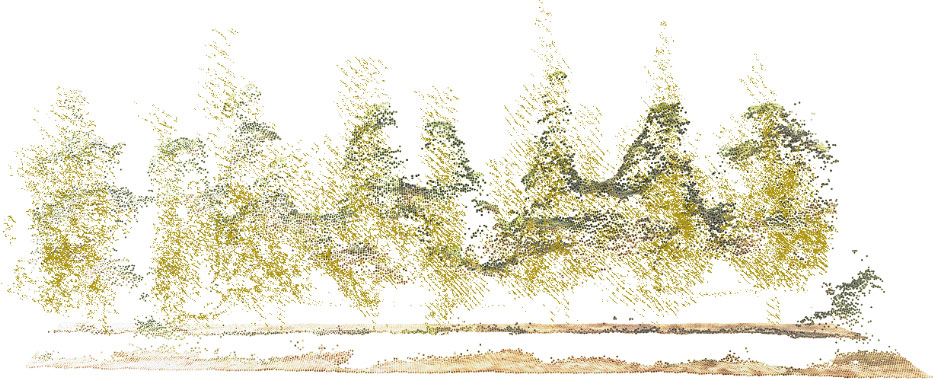
Aligned LiDAR point cloud (yellow) with UAV-constructed DSM (using RGB coloring).

The accuracy of canopy volume measurements did not follow the same order as the accuracy of canopy area segmentation due to multiple sources of error. The UAV-constructed DSM underestimated the canopy height compared to the point cloud acquired by the moving LiDAR (**Figures 9C**, **12**), resulting in underestimated volumes, while the UAV was unable to access the structure of the inner and lower canopy (Brede et al., 2017; Schneider et al., 2019). Other studies have reported underestimates of UAV measurement (Krause et al., 2019; Pourreza et al., 2022). The overhead captured from the UAV resulted in higher leaves obscuring details of the lower canopy, and the reconstructed DSM contained only the upper surface of the canopy, which could lead to an overestimation of volume. The two errors had opposite effects on the volume measurements. With the combination of these two factors, U-Net trained based on DSM alone obtained the best canopy volume measurement with an RMSE of 0.410 m^3^. The RMSE of the UAV-based tree height measurements in this study was 0.644 m. An RMSE of 0.51 m was also obtained in young trees (Vacca and Vecchi, 2024). In a previous study, an RMSE of 0.28 m could be obtained using a high-resolution camera on a flat orchard (Mahmud et al., 2023), and an RMSE of about 0.3 m has been reported in a similar study (Wallace et al., 2016; Birdal et al., 2017; Krause et al., 2019). There is almost only one branch extending upwards at the treetop in this study, while the RGB camera only has 200 million pixels, which affects the accuracy of the reconstruction. There are various ways to measure canopy volume, such as the envelope polygon method and the voxel method. The voxel method was used in this study as a baseline method, and the RMSE of the canopy volume measured with the UAV was 0.410 m^3^. Using the envelope polygon method as a baseline and adjusting the different parameters, the RMSE of measurement is between 0.33 m^3^ and 0.43 m^3^ by the UAV (Mahmud et al., 2023). Based on UAV measurements of canopy volume in apple orchards, the best measurements obtained at different flight heights had an RMSE of 1.41 m^3^, using the ellipsoid fitted by manual measurements as a baseline (Sun et al., 2019). In contrast, the reconstruction process in this study did not use ground control points. With the combination of errors, the accuracy of canopy volume measurements was acceptable.

The canopy measurement process proposed in this study can be used for orchard phenology, pruning, and light interception estimation (Westling et al., 2020; Scalisi et al., 2021). The developed method can obtain the variability of the canopy in spatial distribution and provide prescription maps for precise pesticide spraying, pruning, and other field management work. Compared to LiDAR, the UAV-reconstructed DSM is missing branch details at the treetops, leading to an underestimation of tree height. Also, the ground control point-free reconstruction method may have affected the accuracy of tree height measurement. The UAV-based canopy volume testing process can balance efficiency and accuracy and is particularly suitable for larger orchards.

## 5 Conclusion

This study evaluated the effect of different canopy region segmentation methods on the accuracy of UAV-based canopy volume measurements. RGB and DSM orthophotos constructed based on UAV were used to segment the canopy by U-Net, OTSU, and RANSAC methods and calculate the canopy volume. The results showed that U-Net trained by RGB and DSM achieved the best accuracy in the segmentation task, with 84.75% MIoU and 92.58% MPA. The MPA of segmentation by the OTSU and RANSAC methods is similar to that of the deep learning method, but the MIoU is 65.33% and 64.48%, respectively, which is lower than that of the deep learning method due to the lower overlap of the segmented regions and the obtained canopy mask with a lot of noise. In tree height measurement, the RMSE of tree height measured by LiDAR was 0.430 m, while that of the UAV was 0.644 m. However, the canopy volume measurement task was less affected by the accuracy of tree height measurements. The U-Net trained using only DSM achieved the best accuracy with an RMSE of 0.410 m^3^, an rRMSE of 6.40%, and a MAPE of 4.74%. In contrast, the RMSE of the U-Net segmentation method trained with RGB and DSM was 0.471 m^3^. The canopy volume measurement accuracy of the traditional OTSU and RANSAC methods was lower than that of the deep learning method, with RMSE of 0.521 m^3^ and 0.580 m^3^, respectively. Therefore, in the case of having manually labeled datasets, the segmentation of the canopy region using the deep learning approach can achieve higher accuracy of canopy volume measurement.

## Data availability statement

The raw data supporting the conclusions of this article will be made available by the authors, without undue reservation.

## Author contributions

LH: Conceptualization, Investigation, Methodology, Validation, Writing – original draft. ZW: Conceptualization, Investigation, Writing – review & editing. MH: Conceptualization, Investigation, Methodology, Validation, Writing – review & editing. XH: Funding acquisition, Writing – review & editing.

## Figures and Tables

**Figure 1:**
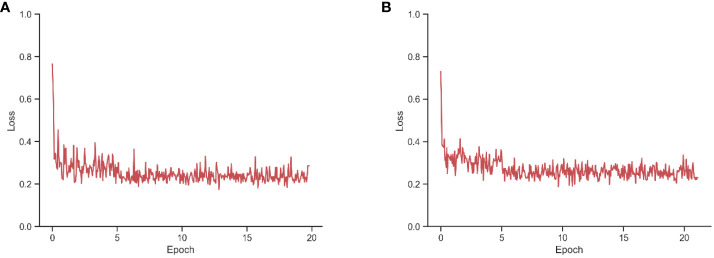
Loss of U-Net network during training with training epoch. **(A)** Trained with RGB and DSM. **(B)** Trained only with DSM.
